# Conrad Fitzgerald

**Published:** 1939

**Authors:** 


### CONRAD FITZGERALD, M.R.C.S., L.S.A.

THE death at the age of 92 in his home at Fortune Bay, Newfoundland, on 2nd February, marks the passing of one who was for many years the senior alumnus of the Bristol Medical School, which he entered in the same year as Dr. Shaw, 1866. He was born at Marlborough, where his father was in practice. At the age of sixteen he was apprenticed to Dr. Lawrence of Bath. After working with him for three years he entered the Bristol Medical School, and was a student at the Royal Infirmary, a prizewinner and the recipient of the Committee's Gold Medal. His biography (published by Messrs. J. W. Arrowsmith Ltd. in 1935, with the title of *The Albatross* from the name of his yacht) gives us many reminiscences of medical apprenticeship and the hospital life of the sixties. He qualified in 1870 and almost immediately began his three years as Ship's Surgeon, during which time he made many voyages to Australia and South America, both in sailing- and in steam-ships. In 1873 he accepted an appointment in Newfoundland, and settled at Harbour Breton in Fortune Bay. He had originally intended to work there for a limited period only, but became so enamoured of the life that he continued there until his death. Here he visited his patients in all weathers in his yacht, the *Albatross* (replaced by a second *Albatross* in 1916), his handling of which became famous throughout Newfoundland. For over sixty years he conducted a practice of the most varied and unexpected sort in an area some forty miles by fifty : when travelling by sea did not serve, it was often a question of many miles in snow-shoes. Burning, drowning, frost-bite, wolf-bite, fractures, epidemics of diphtheria (very dreadful in the pre-antiserum days) all found him ready and well-equipped ; anæsthetics were for long regarded as unnecessary—and in any case were often unobtainable. Fitzgerald was a keen sportsman and regularly recorded his bags of trout, partridge, deer and silver fox. He continued in practice to the end of his life, in the home that was so much part of himself, and died as he had lived, far-famed and greatly loved.

